# Trauma and Hypothermia in Antarctica: An Emergency Medicine Marine Simulation Scenario

**DOI:** 10.7759/cureus.1341

**Published:** 2017-06-12

**Authors:** Chrystal Horwood, Kerry-Lynn Williams, Tate Skinner, Robert Brown, Tia Renouf, Adam Dubrowski

**Affiliations:** 1 Eastern Health, Memorial University of Newfoundland; 2 Faculty of Medicine, Memorial University of Newfoundland; 3 Biochemistry Nutrition, Memorial University of Newfoundland; 4 Offshore Safety and Survival Centre, Marine Institute, Memorial University of Newfoundland; 5 Emergency Medicine, Memorial University of Newfoundland; 6 Emergency Medicine, Pediatrics, Memorial University of Newfoundland

**Keywords:** simulation scenario, emergency medicine, communication skills, trauma, postgraduate medical education, hypothermia

## Abstract

Simulation has been shown to improve both learner knowledge and patient outcomes. Many emergency medicine training programs incorporate simulation into their curricula to provide learners with experiences that are rare to encounter in practice, yet performance with a high degree of competence is critical. One rare encounter, which is depicted in the report, is the management of a trauma patient who was hypothermic after falling from an expedition vessel into the cold Southern Ocean. The unique scenario presented in this technical report incorporates CanMEDS learning objectives including the communicator, health advocate, and collaborator roles. Using medical simulation facilities, marine performance simulation facilities, and a video, this scenario provides teaching that is uncommon in traditional emergency medicine training. As such, it is valuable for trainees who intend to practice rural, remote, or expedition medicine, or provide coverage for ships and marine installations.

## Introduction

Simulation has been shown to improve both learner knowledge and patient outcomes [1]. Many emergency medicine training programs incorporate simulation into their curricula to provide learners with unique experiences incorporating both discipline-specific and CanMEDS learning objectives [2]. As such, the use of non-traditional simulation scenarios can be valuable for trainees who intend to practice rural, remote, or expedition medicine, or provide coverage for ships and marine installations.

Resource management is challenging in a limited environment such as a ship’s infirmary on the many passenger ships/cruises that frequent Antarctica. Antarctica is the coldest, windiest, and driest continent in the world [3]. It is difficult to provide patient care in the oceans that surround it. Still, tourists and researchers travel there in ever-increasing numbers. Ships’ logs report lists of accidents and injuries that are encountered in the region [4]. Based on this list, we have developed a trauma/hypothermia scenario that takes place in the pre-hospital setting aboard a ship off the coast of Antarctica. The scenario provides fundamental learning objectives that the learner should be able to attain upon completion. These objectives are as follows:

1. Assess and manage trauma in a limited marine environment 2. Considerations for transport to a more stable environment 3. Communicate with the Captain in preparation for transport to a land-based centre

From these objectives, the learner should gain valuable clinical pearls that are intrinsic to emergency medicine training. This scenario, while rare, provides simulated teaching and learning in an extremely challenging pre-hospital setting. This is important for learners who, after graduation, may practice in remote and rural settings that may greatly affect the level of care they are able to provide.

## Technical report

Providing emergency medical treatment onboard ships at sea can be difficult, depending on the severity of the injury, the available resources in the ship's infirmary, and the physician's experience. Ships transporting goods by sea generally carry a small number of trained crew-members, while large cruise ships have a mixture of seafaring crew, hotel crew (typically only minimum seafarer training), and passengers in large numbers (totaling over 8,000 for the largest ships). For the former, the onboard medical provider is usually the ship’s cook with advanced first aid training, while the latter may carry one or more physicians with a small team of nurses and a well-equipped onboard hospital.

Smaller expedition-style cruise ships traveling in remote polar regions tend to have just one ship’s doctor with a small infirmary. There is little advanced resuscitation equipment and often no investigative resources, a reality that challenges physicians who are trained to work in urban emergency departments [5].

Injured passengers and crew, on smaller vessels, face greater challenges in receiving emergency medical treatment than on large ships in less remote areas. This is related to the extreme polar environmental conditions, long distances to better-equipped health care facilities, and communications. In a medical emergency on land, the attending physician is in charge. At sea, however, medical management occurs within limits established by the Captain, as patient care is determined by factors including sea state, rescue options, and clinical stability. This unusual dynamic requires solid communication skills, as demonstrated in the learning objectives [6].

Given the impact this unique environment can have on the level of care provided, we decided to develop this scenario to utilize both a medical simulation lab and a marine simulation centre. We hope this realism will illustrate the limitations associated with marine polar environments, and the challenges of stabilizing a significantly ill patient for an extended period of time in a nontraditional environment. Many sites may not have access to a marine simulation centre. Therefore, this scenario can also be used in a standard simulation lab, using the video footage presented in this technical report in order to re-enact the patient’s fall into the Southern Ocean, his extraction from the water, and his transfer to the medical facility onboard the ship. The learner could then continue the simulation case in a standard simulation lab, beginning at the examination/management stage, using a high-fidelity manikin, a standardized patient, and confederates. The transfer conversation between physician and captain could also be simulated in a medical simulation lab.

Pre-Briefing

Prior to the start of the case, a formal pre-briefing session was held with all learners present. This session is a vital component in any simulation scenario as it sets the learner/teacher interaction. The purpose of the pre-briefing session is to establish a safe learning environment. It also introduces the fictional contract, which states the learner agrees to proceed as if the simulation was real while simultaneously acknowledging that it is not. Limitations of the simulation were also addressed, with particular attention to technical issues with the mannequin and the environmental setup. Finally, learners were advised that they would not be formally evaluated.

Prior to the session, a detailed scenario template and corresponding storyboard were developed and submitted to the medical and marine simulation labs technical staff (Table 1). A script was also prepared, based on the patient’s history and injuries, and submitted to the standardized patients in preparation for the scenario.

Two instructors were present during the simulation: one maintained overall control of the scenario, and the other observed and prepared for the subsequent debriefing. 

**Table 1:** The scenario template that was submitted to the simulation lab’s technical staff in advance of the scenario ABCDE - Airway, breathing, circulation, disability, exposure BP - Blood pressure C-spine - Cervical spine GCS - Glasgow coma scale GERD - Gastroesophageal reflux disease HR - Heart rate ID - Identification IV - Intravenous L - Litres NP - Nasal prongs RA - Room air RR - Respiratory rate T - Temperature VS - Vital signs

Scenario		
You are a physician working on a tourism vessel operating out of Antarctica. You receive a call about an injury sustained by a crew-member while working. You receive the call on the ship, and the patient is rescued and brought to you via an inflatable zodiac boat. Manage and treat this patient.		
Begin scenario – Learner answers the page, arrives on scene in a Zodiac boat		
Objective 1: Considerations for transport to a more stable environment		
Additional data/findings	Vitals	Appropriate Learner Action
While working on a tourism vessel, a 56-year-old male crew-member is tightening a cable and it lets go, causing a metal beam to swing toward him. Although he tries to move out of the way, the beam hits him in the abdomen and he is pushed overboard. He is wearing a life jacket.	GCS 11	Takes initial history: Events of incident Signs/Symptoms Allergies Medications Past medical history Last ate
History		
Events of incident	As above	
Signs/Symptoms	Contusion/blood on the back of the head. The patient is cold, wet, and not shivering. Slight blue discoloration around the mouth.	
Allergies	Environmental	
Medications	Omeprazole	
Past medical history	GERD	
Last ate	5 hours ago (eggs, toast, and tea)	
Recognizes the potential for C-spine injury and hypothermia	Stabilizes C-spine and the patient is back-boarded before transportation to the ship’s medical centre. Requests any available blankets.	
Objective 2: Assess and manage trauma in a limited environment		
*This objective starts once the patient is in the ship’s medical centre*		
Expected Actions		
Remove all wet clothes, get a full set of vitals, including glucose and temperature. Obtain IV access, and attach to oxygen saturation and cardiac monitors.		
Additional data/findings		
Initial vitals	BP 98/64 HR 47 RR 9 O2 87-89% RA T 32.2 GCS 11 The patient is opening eyes to painful stimuli, is confused and drowsy and localized to pain. C-spine precautions are being followed. Learner should continue with ABCDE assessment	
Airway	The patient is able to speak but is slow and confused. The patient is collared and back-boarded.	
Breathing	Coarse breath sounds heard bilaterally. Respiratory effort low.	
Circulation	Cool skin, pale, and wet. Slight blue discoloration around the lips. The abdomen is tender in the left upper quadrant with some bruising noted. Left shoulder pain. No obvious long bone fractures or deformities	
Disability	Pupils equal and reactive to light, accommodation normal. GCS 11 confused and drowsy, opening eyes to painful stimuli and localizing to pain. Moving all limbs.	
Exposure	Laceration to the right occipital lobe, swelling and bruising. Pupils equal and reactive to light, accommodation. Log-rolled, no obvious trauma findings or deformities, no spinal tenderness.	
Additional data/findings	Vitals	Appropriate Learner Action
The learner must clean and dress the wounds.	Update VS after wounds attended and secondary survey. BP 95/60 HR 54 RR 11 O2 96% 2L NP T 31.9 GCS 13-14	Log-roll/secondary survey looking for other injuries. There will be no additional findings on the secondary survey. Start warmed IV fluids and active rewarming. If the learner does not call the Captain/coastguard, have the phone ring.
Objective 3: Prepare for transport to a tertiary care centre		
Provide a verbal report to the captain.		Report should include: - ID - Mechanism of injury - Events of injury - Signs/symptoms - Differential diagnosis - Last vitals - Interventions *Example:* 56-year-old male thrown overboard when accidentally struck on the left side with a metal beam. He was in the water for 7 minutes when the rescue crew was able to haul him out. He has since been transferred to the medical facility on the ship and assessed. He is experiencing symptoms consistent with significant hypothermia and potential intra-abdominal trauma. States last vitals, and lets them know he is being actively re-warmed and getting warm IV fluids. Given his extensive injury, he needs to be transferred to a tertiary care centre.

Case

This case began with the learner receiving a distress call on a ship. The learner was a physician working on a tourism vessel operating out of Antarctica. The distress call detailed an injury sustained to a crew-member who was hit by a cable while working, and fell overboard. The learner received the call on the ship and was expected to manage and treat this patient.

A video showing a complete run-through of the scenario is included. It is intended to provide a step-by-step approach to the case (Video 1).

**Video 1:** Trauma and hypothermia off the coast of Antarctica: an emergency medicine marine simulation scenario This simulation used a Laerdal SimMan 3G™ as the high-fidelity manikin

Debriefing

At the end of the scenario, a structured debriefing session was held. The debriefing was lead by an educator with experience in debriefing. The principles of good judgment and frame discovery were central to the process. The purpose of the session was for the learners to interact with the educators and to review the entire case, areas of concern, and specific learning topics. Immediate review of the case in a small, safe, and relaxed environment encouraged the learner to speak freely and gain the most from the experience. Points included in the debriefing session are included in Table 2.

**Table 2:** Debriefing/guided reflection questions

How did you feel throughout the simulation experience?
What went well in this simulation?
What did not go well in this simulation?
Were you satisfied with your ability to work through the simulation?
If you were able to do it again, how could you have handled the situation differently?
How did the limited resources impact your management of the situation?
Do you think that the considerations for transport to a tertiary centre vary much between land and sea? Why or why not? If yes, how?
Is there anything else you would like to discuss?

Post Scenario Didactics

Predetermined education topics were discussed in the post-scenario didactic session. The didactic section allowed the educator to address individual knowledge gaps that were recognized throughout the simulation. Here, the educator was able to provide individualized teaching to help solidify new knowledge gained throughout the scenario. Points covered in the didactic session included principles of trauma management, effective communication, transport considerations, and handover. Learners were asked to identify ways to improve communication and inter-agency collaboration when preparing for transport.

## Discussion

This scenario was designed to challenge postgraduate emergency medicine learners with a non-traditional trauma scenario. In this simulation, key learning objectives for trainees included:

1. Assess and manage trauma in a limited marine environment 2. Considerations for transport to a more stable environment 3. Communicate with the Captain in preparation for transport to a land-based centre

These learning objectives demonstrate that the environment in which the scenario is carried out plays an important role in the patient’s outcome. Trauma generally occurs outside typical emergency department settings; one has to work with what they have to stabilize a patient, know their capabilities and limitations, and arrange for transport to a higher-level facility.

The learner must recognize the need for transfer and appreciate that in remote regions, conditions may be prohibitive. For example, the weather might impede air evacuation. In polar regions like the Antarctic Peninsula (where most small expeditions travel), the nearest evacuation centre may be a small research station that has one physician. Research centres are not mandated to accept patient transfers from tourist expeditions, but in the spirit of international cooperation will generally do so in an emergency. Some airstrips in Antarctica are usable only in winter when the ice is stable. However, most small ship tours operate only in summer [7]. In these situations, the learner must realize their limitations while stabilizing a patient until the transportation is made possible. Such scenarios are not often encountered during a typical emergency medicine residency, as most training occurs in a tertiary academic setting.

Simulation has been used for decades as a tool to assess and strengthen various skills including communication, decision-making, leadership, task management, and patient monitoring. The benefits of simulation training are immediate formative feedback, ability to re-practice the scenario, and alter the level of difficulty for various learners [8].

The scenario developed in this case employed two types of simulation: medical and marine simulation augmented with a demonstrative video. One of the learning objectives was trauma management in a remote setting using limited tools. A marine simulation centre was therefore needed to develop realistic sea states, winds, waves, and ice to emphasize the environmental difficulties that could be encountered in such real life settings. While the entire case could have been executed in a medical simulation centre, using a standard mannequin and assuming there had been a trauma in cold water, much environmental significance would have been lost.

This scenario was recorded because access to a marine simulation centre is not commonly available. Instructors may play the recorded video either before the learner begins the examination, or afterwards during the debriefing session. The pre-recorded video will allow the learner to see and hear the real environment and its significance to the case.

Concomitant use of both kinds of simulation, augmented with a demonstration video, should enhance the learner’s experience of a unique scenario whose remote setting and harsh marine environment were essential to learning objectives. Such scenarios are not commonly encountered in the traditional tertiary care simulation syllabus. The experience is potentially valuable for rural and remote practising physicians and those who provide medical coverage for ships and oil installations, as well as for expedition physicians.

## Conclusions

Trauma resuscitations are challenging, particularly in remote marine environments. We present a marine trauma simulation that uses medical and marine simulation centres, and a video. It provides valuable training for those who intend to practice rural, remote, or expedition medicine, or provide coverage for marine ships and installations. It allows for the individuals to grasp the impact one’s environment can have on the practice of medicine and patient outcomes.
